# Pseudocirrhosis in Chronic Budd Chiari Syndrome With Janus Tyrosine Kinase 2 (JAK2) Mutation

**DOI:** 10.7759/cureus.9355

**Published:** 2020-07-23

**Authors:** Seetha Lakshmanan, Dhanya Baskaran, Yashvin Onkarappa Mangala, Nabil Toubia

**Affiliations:** 1 Internal Medicine, Roger Williams Medical Center, Boston University School of Medicine, Providence, USA; 2 Internal Medicine, Geriatric Research, Education and Clinical Center, Bruce W. Carter Veterans Affairs Medical Center, Miami, USA; 3 Internal Medicine, Roger Williams Medical Center, Providence, USA; 4 Gastroenterology, Roger Williams Medical Center, Providence, USA

**Keywords:** cirrhosis, jak2 mutation, budd-chiari syndrome

## Abstract

Budd-Chiari syndrome (BCS) occurs when there is hepatic venous outflow obstruction. Chronic BCS may result in liver cirrhosis due to long-standing obstruction and tend to present late. We present the first case of BCS secondary to Janus tyrosine kinase 2 (JAK2) mutation resulting in "pseudocirrhosis" rather than cirrhosis of the liver. Pseudocirrhosis clinically and radiologically mimics cirrhosis without the classical histopathological changes, and it is usually associated with metastatic cancers.

## Introduction

Hepatic venous outflow obstruction from hepatic veins to the superior end of the inferior vena cava may lead to a rare disorder called Budd-Chiari syndrome (BCS). BCS may present differently, from complete absence of symptoms to fulminant hepatic failure, depending on the location, severity of obstruction, and presence of collaterals [1]. The disease course may be acute, subacute, or chronic with the development of symptoms ranging from weeks to months before the diagnosis is made. Myeloproliferative disorders, especially the V617F Janus tyrosine kinase 2 (JAK2) mutation, account for about 40% to 60% of these patients [2]. Herein we present a case of BCS secondary to JAK2 mutation resulting in pseudocirrhosis of the liver.

## Case presentation

**Figure 1:**
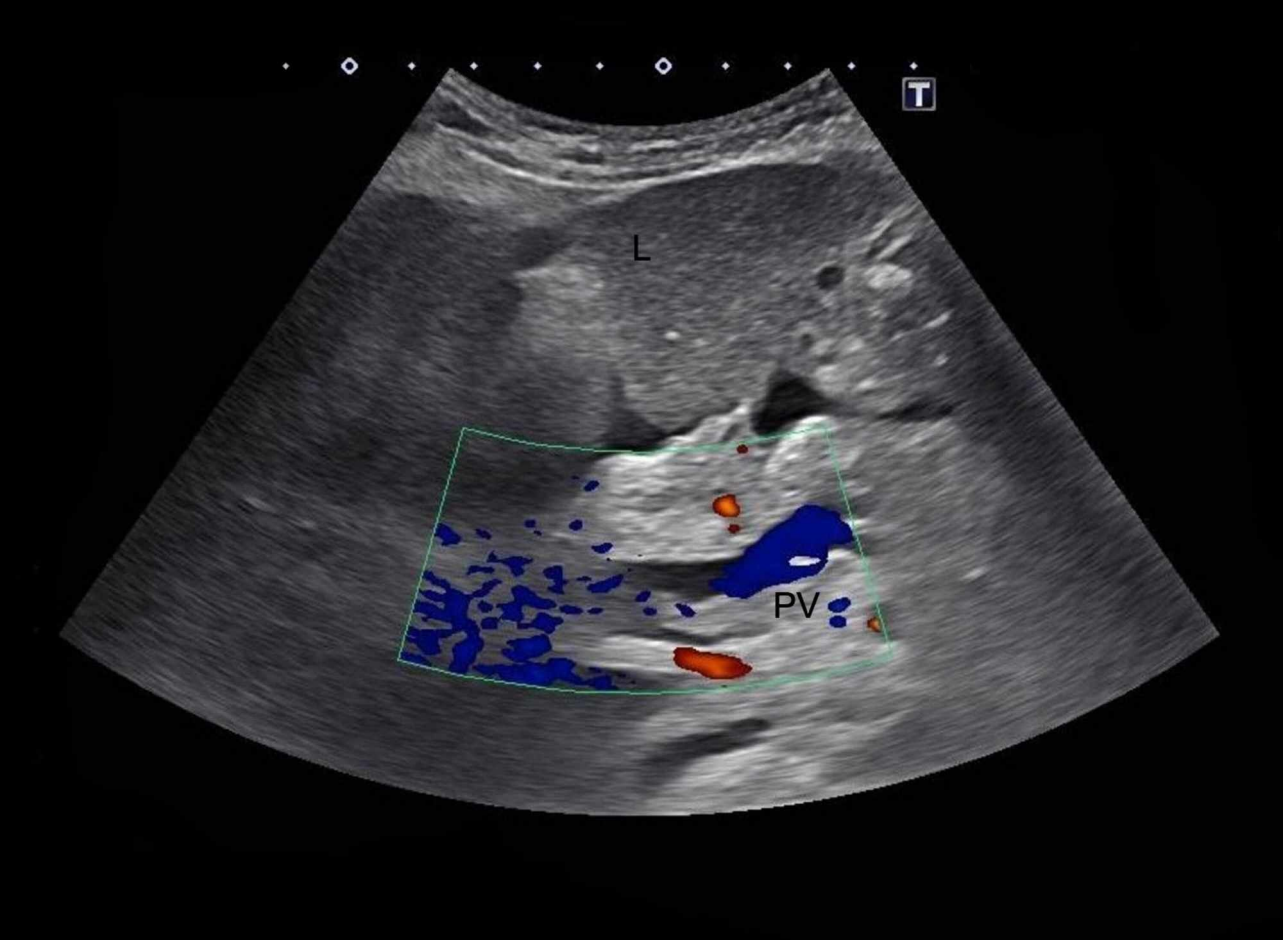
Ultrasound of the abdomen revealing an apparent portal vein thrombosis of extrahepatic portion of the portal vein (PV), identified anterior to the pancreatic head as demonstrated by lack of color on Doppler ultrasound.

A 74-year-old female patient with hypothyroidism and a remote history of alcoholism presented to our facility with bleeding per rectum for two days. She denied any family history of malignancy, tobacco or substance use, or prior endoscopic evaluation. On admission, the patient was hemodynamically stable and her physical examination was benign. Initial laboratory workup revealed white blood cell count (WBC) 18,400/µL, hemoglobin 16.8 g/dL, platelet 588,000/µL, aspartate aminotransferase (AST) 570 U/L, alanine aminotransferase (ALT) 804 U/L, alkaline phosphatase (ALP) 126 U/L, and total bilirubin of 3.6 mg/dL with a direct bilirubin of 1.0 mg/dl and indirect bilirubin of 2.6 mg/dL. Hepatitis panel was negative. Ultrasound of the abdomen with Doppler revealed ascites and portal vein thrombosis (Figure 1). Follow-up MRI of the abdomen confirmed a non-occlusive thrombus in the main portal vein and non-opacification of the major intrahepatic veins, both suggestive of BCS. There was also diffuse heterogeneity of the liver parenchyma without focal mass and large ascites with bilateral pleural effusions. 

**Figure 2:**
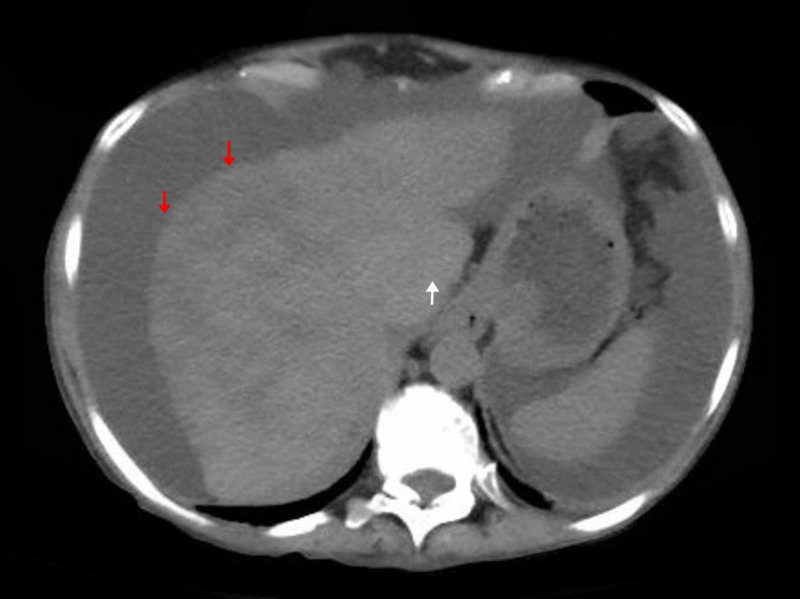
CT of the abdomen done after three months of diagnosis revealing cirrhotic morphology with nodular shrunken liver (red arrows), caudate hypertrophy (white arrow), and diffusely heterogeneous attenuation.

The patient was worked up for BCS starting with a full hypercoagulability panel and only JAK2 mutation was positive. Diagnostic paracentesis revealed transudative ascitic fluid without evidence of malignancy, while liver elastography confirmed the presence of cirrhosis. Esophagogastroduodenoscopy (EGD) revealed grade 2 esophageal varices, while colonoscopy was unremarkable. Echocardiogram, on the other hand, showed an ejection fraction of 65% with normal ventricular function. All other possible etiologies of congestive hepatopathy or cirrhosis were excluded. The patient was subsequently started on intravenous heparin for the thrombosis and later transitioned to daily oral rivaroxaban prior to discharge. Over the next three months, the patient required recurrent hospitalizations for decompensated liver disease and underwent biweekly therapeutic paracentesis to relieve her symptoms (Figure 2).

**Figure 3:**
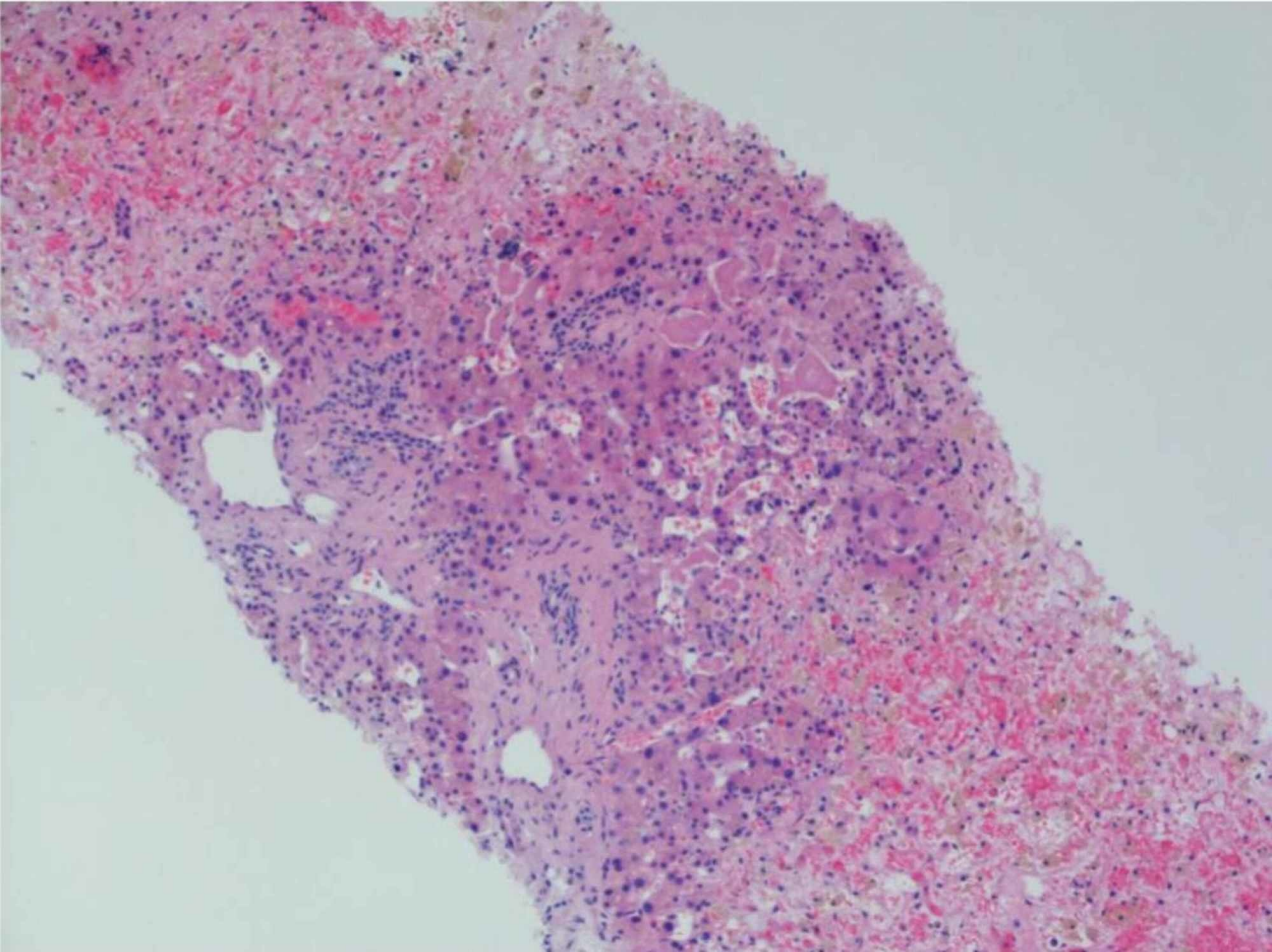
Liver biopsy with hematoxylin and eosin stain shows centrilobular hepatic cell plate necrosis without evidence of nodular regenerative hyperplasia and periportal residual viable hepatic cell plates. These findings are consistent with hepatic outflow obstruction in the absence of cirrhosis.

During her last admission, however, we noted that she had preserved synthetic liver function and inconsistencies in her presentation. A liver biopsy was then obtained which revealed centrilobular hepatocellular necrosis, sinusoidal dilatation, and congestion with mild portal fibrosis consistent with hepatic outflow obstruction. Hematoxylin and eosin stain highlighted the centrilobular collapse/hepatic necrosis (Figure 3). However, there was no evidence of nodular regenerative hyperplasia or cirrhotic findings. Her “pseudocirrhosis” was concluded to be a result of the splanchnic venous thrombosis. She was later transferred to an advanced facility for portal vein reconstruction or plasty to relieve the hepatic outflow obstruction and reverse the pseudocirrhosis. Unfortunately, the patient developed severe hepatorenal syndrome and hypovolemic shock from variceal bleeding there. She eventually underwent palliative direct intrahepatic portosystemic shunt procedure and was discharged home on hospice leading to her eventual demise.

The abstract of this article has been presented at the American College of Gastroenterology Conference, 2019 in San Antonio, Texas [3].

## Discussion

Approximately 20% of BCS patients usually remain asymptomatic and present with spontaneous development of large intrahepatic and portosystemic collaterals [4]. It is also not uncommon to see patients suffering from recurrent ascites and gastrointestinal bleeding before finally diagnosed as BCS. Cirrhosis caused by chronic BCS manifests as generally preserved liver function, though clinically they present with portal hypertension, ascites, and variceal bleeding. Peng et al. studied the imaging features of collateral circulation on CT and demonstrated that caudate lobe enlargement and intra or extrahepatic collaterals are more common in cirrhosis caused by BCS (73%) compared to hepatitis B infection (8%) [5].

Pseudocirrhosis clinically and radiologically mimics liver cirrhosis without the classical histopathological changes seen in cirrhosis. Liver biopsy is the key for diagnosis, confirming the absence of typical regenerating nodules of hepatocytes or fibrotic bridges between the nodules as seen in cirrhosis. Pseudocirrhosis is often associated with metastatic cancers and is typically seen in breast cancer patients with liver metastasis receiving systemic chemotherapy. The pathophysiology is believed to be in response to chemotherapy-induced ischemic injury [6]. However, no single chemotherapy agent has been identified to cause this and there are no proper guidelines in place for prevention. If pseudocirrhosis is identified, temporarily holding the chemotherapy agent and supportive management may lead to an improvement in clinical symptoms. 

To our knowledge, this is the first reported case of pseudocirrhosis in BCS. Unlike advanced cancers whereby systemic chemotherapy is essential mandating treatment despite the known complication, pseudocirrhosis in BCS can be prevented when identified early and intervened to reverse the disease. Apart from anticoagulation, these patients may require other interventions to bypass the obstruction. Direct rather than transjugular intrahepatic portosystemic shunt placement would be appropriate due to the hepatic vein thrombosis resulting in difficulty to catheterize the hepatic veins.

## Conclusions

BCS occurs when there is hepatic venous outflow obstruction. Chronic BCS may result in liver cirrhosis due to long-standing obstruction and tend to present late. We present the first case of BCS secondary to JAK2 mutation resulting in "pseudocirrhosis" rather than cirrhosis of the liver. Pseudocirrhosis clinically and radiologically mimics cirrhosis without the classical histopathological changes, and it is usually associated with metastatic cancers.
